# Current Devices in TMVI and Their Limitations: Focus on Tendyne

**DOI:** 10.3389/fcvm.2020.592909

**Published:** 2020-12-23

**Authors:** Gry Dahle

**Affiliations:** Oslo University Hospital, Oslo, Norway

**Keywords:** TMVI, Tendyne, patient selection, challenges, planning, complications

## Abstract

Mitral valve regurgitation (MR) has a high incidence in the western world, and mortality is high for untreated severe MR. Catheter based repair was introduced with MitraClip in 2003, and some additional devices later came into the market. To expand the transcatheter treatment options for mitral valve disease, the first transcatheter mitral valve implantation (TMVI) was performed by Søndergaard et al. 2012, only 10 years after the first transcatheter aortic valve implantation (TAVI), however, the development has been much slower for the TMVI than for TAVI. From 2012, studies were started for several devices to prove feasibility and safety. However, there were big challenges in valve design; delivery systems and anchoring in addition to anatomical issues (avoid LVOT obstruction and paravalvular leak, big size of annulus). The main valves in studies were CardiaQ (later bought by Edwards Lifesciences, Irvine, United States), Tiara (Neovasc Inc., Richmond, Canada), Twelve (later Intrepid, Medtronic, MN, United States) and Tendyne™ (Abbott, MN, United States). I will focus on the Tendyne™ valve that is the only CE approved transcatheter mitral valve implant. It is available in a large number of sizes and is repositionable and retrievable. The results for the 100 first patients included in the early feasibility study (EFS) at 1 and 2 years are promising. Initially feasible for MR, but further investigations show promising results also for implant in mitral annular calcification.

## Introduction

The mortality rate for untreated severe mitral regurgitation (MR) is up to 50% at 5 years (1, 2), and the incidence in the western world of such patients is 1–2%, with a prevalence of 10% for patients >75 years (3). Surgical repair and replacement have for a long time been the standard treatment option for this condition, but unfortunately this has not been offered to a large number of patients. The transcatheter mitral valve repair, first with the MitraClip (Abbott Vascular, Abbott Park, IL, United States), was introduced in 2003, and had CE mark in 2008. More recently, other repair devices have also come to the market, like NeoChord (NeoChord Inc, MN, United States), Cardioband (Edwards LifeSciencse, Irvine, United States), Carillon (Cardiac Dimensions INC, WA, United States) and Pascal (Edwards Lifesciences, Irvine, United States) (4). However, not all pathologies are repairable, even with these additional repair device strategies. To expand the transcatheter treatment options for mitral valve disease, the first transcatheter mitral valve implantation (TMVI) was performed in 2012 with the CardiAQ valve (Edwards Life Sciences, Irvine, California, United States) (5). This event was 10 years after the first transcatheter aortic valve implantation (TAVI), as development has been much slower for TMVI systems. There are several reasons for this slower development:

1. Mitral regurgitation is a heterogeneous disease
2. Repair is generally the preferred surgical treatment option, though this is highly dependent on the experience of the center and about 2/3 of mitral valve surgery is repair (6); therefore, the question is if the same strategy should be used for transcatheter treatment
3. The mitral valve anatomy is much more complex than the aortic valve
4. Additional pathologies are common in patients with mitral valve disease such as aortic stenosis, tricuspid regurgitation, left ventricular dys-synchrony, atrial fibrillation or heart failure. These have to be addressed in addition to the replacement or repair the mitral valve. Generally, aortic valve disease, resynchronization therapy, atrial fibrillation and heart failure treatment are addressed before proceeding with mitral intervention. If needed, the tricuspid valve is treated after the mitral valve
5. The durability of bioprostheses in the mitral position is questionable

**Figure 1:**
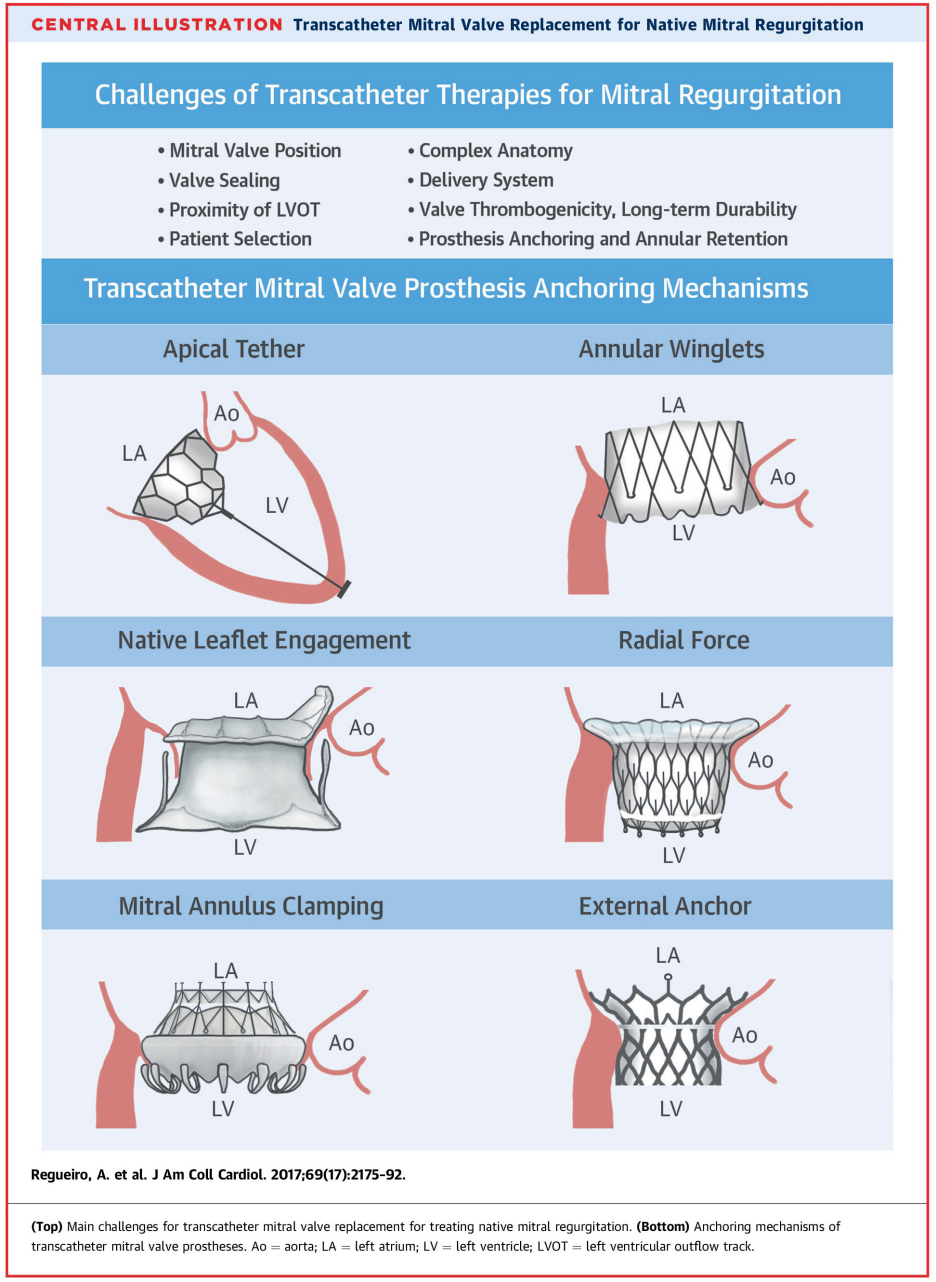
Different anchoring mechanisms for catheter valves in mitral position. Please see text. From Central illustration, Regueiro et al. (7).

**Figure 2:**
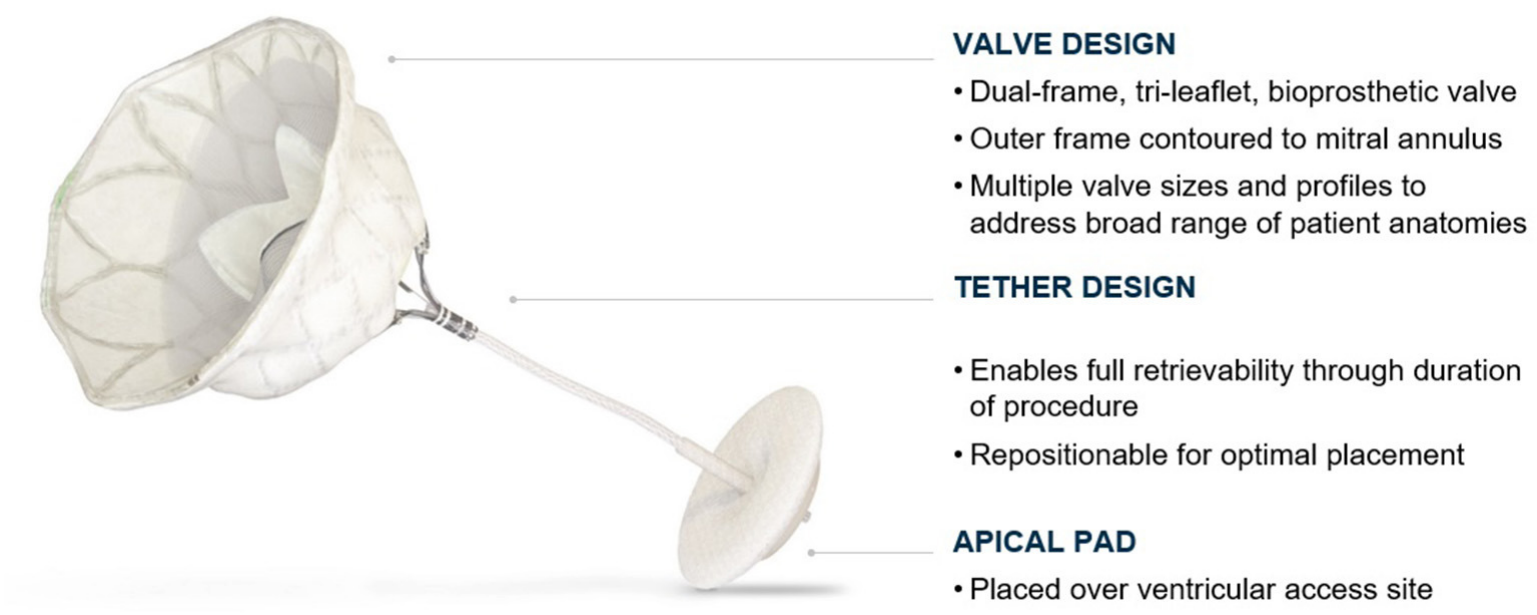
Tendyne™ valve. The Tendyne valve is a tri-leaflet porcine bioprosthetic valve with an outer and inner frame. The valve is anchored by a tether secured by an apical pad. Courtesy of Abbott.

Several TMVI systems are in clinical studies for human implant (4), with different properties and design. The four valves with the most clinical experience are the CardiAQ, Tiara (Neovasc, Richmond, BC, Canada), Tendyne™ (Abbott Medical, St Paul, MN, United States) and Intrepid valves (Medtronic, Minneapolis, MN, United States), Table 1 and Figure 1. In this article, we focus on the Tendyne™ valve, which is a tri-leaflet porcine bioprosthetic valve with a circular inner frame and an outer frame contoured to fit the mitral annulus, anchored to the apex with a tether and apical pad, Figure 2. The only TMVI system with CE approval is the Tendyne™ (Abbott Medical, St. Paul, MN, United States). The approval was achieved in January 2020, Figure 3. More than 400 valves have been implanted in the clinical experience to-date, and results of the first 100 patients with Tendyne™ at 1 year (8) and 2 years, (9) are very promising, with 96% technical success per MVARC definition, 30 day mortality of 6%, and one-year survival of 72.4%.

**Table 1:** Summary of the properties for the four transcatheter mitral valves with most experience.

**Property**	**CardiAQ edwards**	**Tiara neovasc**	**Tendyne abbott**	**Intrepid medtronic**
Valve shape	Circular	D-shaped	Outer frame contoured to mitral annulus Circular inner frame	Circular
Frame	Nitinol, self-expandable	Nitinol, self-expandable	Nitino, double stent, Self-expandable	Nitinol, double stent, Self-expandable
Anchoring mechanism	Mitral annulus capture with native leaflet engagement	Fibrous trigonum capture with native leaflet engagement	Apical tether	Radial force and subannular cleats
Leaflets	Trileaflet, Bovine pericardium	Trileaflet, Bovine pericardium	Trileaflet, Porcine pericardium	Trileaflet, Bovine pericardium
Access	Transapical, transseptal	Transapical	Transapical	Transapical
Valve position	Supra-annular	Intra-annular	Intra-annular	Intra-annular
Delivery sheath size	33 F	32 F	36 F	35 F
Recapture/Resheathable	No	No	Yes	No
Valve sizes	30mm	35 and 40mm	Outer frame in 8 standard and 5low profile, 2 inner frame sizes, see Figure 6	27 mm with 3 outer stent sizes: 43, 46, and 50 mm
Additional features	Supra annular position, intra annular sealing skirt, tapered outflow	2 anterior and 1 posterior anchoring structures	2 inner frame sizes, 13 outer frame sizes, standard and low profile	Dual stent design, outer frame provides fixation and isolates the inner stent

**Figure 3:**
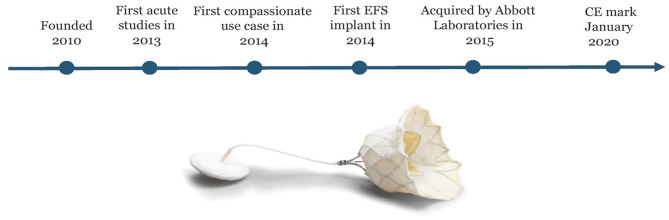
The history of Tendyne: The company was founded in 2010. First implant done in acute studies in 2013 and first compassionate use case in 2014 before first EFS case was done in 2014. In 2015 Tendyne was acquired by Abbott Laboratories. The Tendyne system became the first TMVI system to receive CE Mark approval in January 2020. Courtesy of Abbott.

## Challenges In Valve Design

- The TMVI prosthesis frame must be able to be crimped down and conform to a low-profile delivery system, and on expanding from the delivery system, the frame “remembers” its shape before crimping.
- The valve must withstand the dynamic pressure and flow conditions prevailing within the left ventricle during systole and diastole. The design must additionally have an anchoring system that maintains the valve in place throughout these dynamic conditions after final placement.
- Minimizing outflow tract obstruction and allowing for the maximum amount of blood flow through the left ventricular outflow tract is vital for the patient's heart function.
- Proper blood flow washout to avoid flow stagnation is important to prevent thrombosis, especially for mitral prostheses which are larger, resulting in more synthetic material implanted, and partially reside in the atrium with low flow velocities.
- Proper conformation with optimal sealing prevents paravalvular leakage (PVL) and resultant turbulent flow which can cause thrombus formation or hemolysis.
- Closely matching the natural shape of the mitral annulus can improve valve performance and reduce PVL.
- A design that can be fully repositioned and retrieved during the initial implant procedure allows for optimal valve placement and can mitigate outflow tract obstruction.

## Challenges In Anchoring

The mitral annulus is d-shaped and in regurgitation offers little support. The fixation of the prosthesis may therefore be challenging, and migration of the valve before sufficient tissue ingrowth provides stability is a concern because of the high pressures during the systolic phase when the valve is closed. The valve generally needs to be seated within non-calcified tissue that is both dynamic and D- shaped in one plane and saddle shaped in three dimensions. In some cases, mitral annular calcification (MAC) is present and presents distinct challenges due to the heterogeneous mechanical properties and geometry of the annulus.

The anchoring systems utilized by current TMVI systems include:

- A tether and epicardial pad to achieve counteracting axial forces, Tendyne™ valve (8)
- Native leaflet grasping to fixate the prosthesis in place, Tiara valve (7)
- Docking system to allow radial forces sufficient enough for fixation, HighLife (Highlife Medical, Irvine, California) (7) Sapien M3 (Edwards Lifesciences, Irvine, California) J Webb, TCT 2019
- Atrial and ventricular flanges to grasp the mitral valve annulus and leaflets, CardiAQ (7)
- Sub annular hooks that pierce the native mitral valve tissue/annular winglets, NaviGate (NaviGate Cardiac Structures, Lake Forest, California) (7)
- Cork-like effects that produces radial forces to aid the anchoring of the prosthesis, Intrepid valve (7)
- Atrial cages that use the full anatomy of the left atrium to prevent valve migration, AltaValve, 4C Medical, Marple Grove, Minneapolis, Minnesota, United States) (10)

## Pre-Operative Planning

The approved indications or clinical study eligibility criteria have to be met for treatment with TMVI. Normally, these criteria include an ejection fraction above 30% and a left ventricular diastolic diameter <7.0 cm. The regurgitation should be more than 2+ and the patient should be symptomatic to motivate treatment. Both primary and secondary MR etiologies can be addressed with TMVI.

Echocardiographic evaluation of the severity of mitral regurgitation, length of anterior leaflet and presence of systolic anterior motion (SAM), resulting in hemodynamic challenges, should be reviewed when selecting patients for TMVI. The function of the non-mitral valves, heart rhythm and ejection fraction must also be addressed.

CT reconstruction has to be done for

- Prosthesis sizing
- Calcium in annulus
- Evaluation of neoLVOT (aorto mitral angle, septal bulge, anterior leaflet) (11)
- Thickness of myocardium and papillary muscle anatomy
- Implantation angles for best coaxiality
- Chest access

The 3Mensio (Pie Medical Imaging, Maastricht, Netherland) Materialize (Loeven, Belgium) and Circle CVI (Cardiovascular Imaging Inc, Calgary, Canada) software have all been used for the CT- reconstructions, and in some cases have permitted 3D printing. Of great importance is that the CT scan is done with thin slices (0.5–0.75 mm), ECG-gated and contrast-enhanced, and across the entire heart cycle. The entire chest also should be analyzed with a non-contrast CT for transapical access planning (site and coaxiality). The software should have the ability to simulate the valve of the planned size in the annulus to calculate the predicted neoLVOT. Identifying the access site on the left ventricle, the implantation trajectory and the relation to the native apex is crucial to achieve the best coaxiality as current transapical TMVI delivery systems are not steerable.

The anatomy of the papillary muscles is mapped to avoid damaging them in in the access.

The calcium in the annulus and leaflets is evaluated for potential hazards. Excessive calcium in the leaflets may be prohibitive without adjunct procedures such as balloon valvuloplasty to permit an optimal placement of the prosthesis.

**Figure 4:**
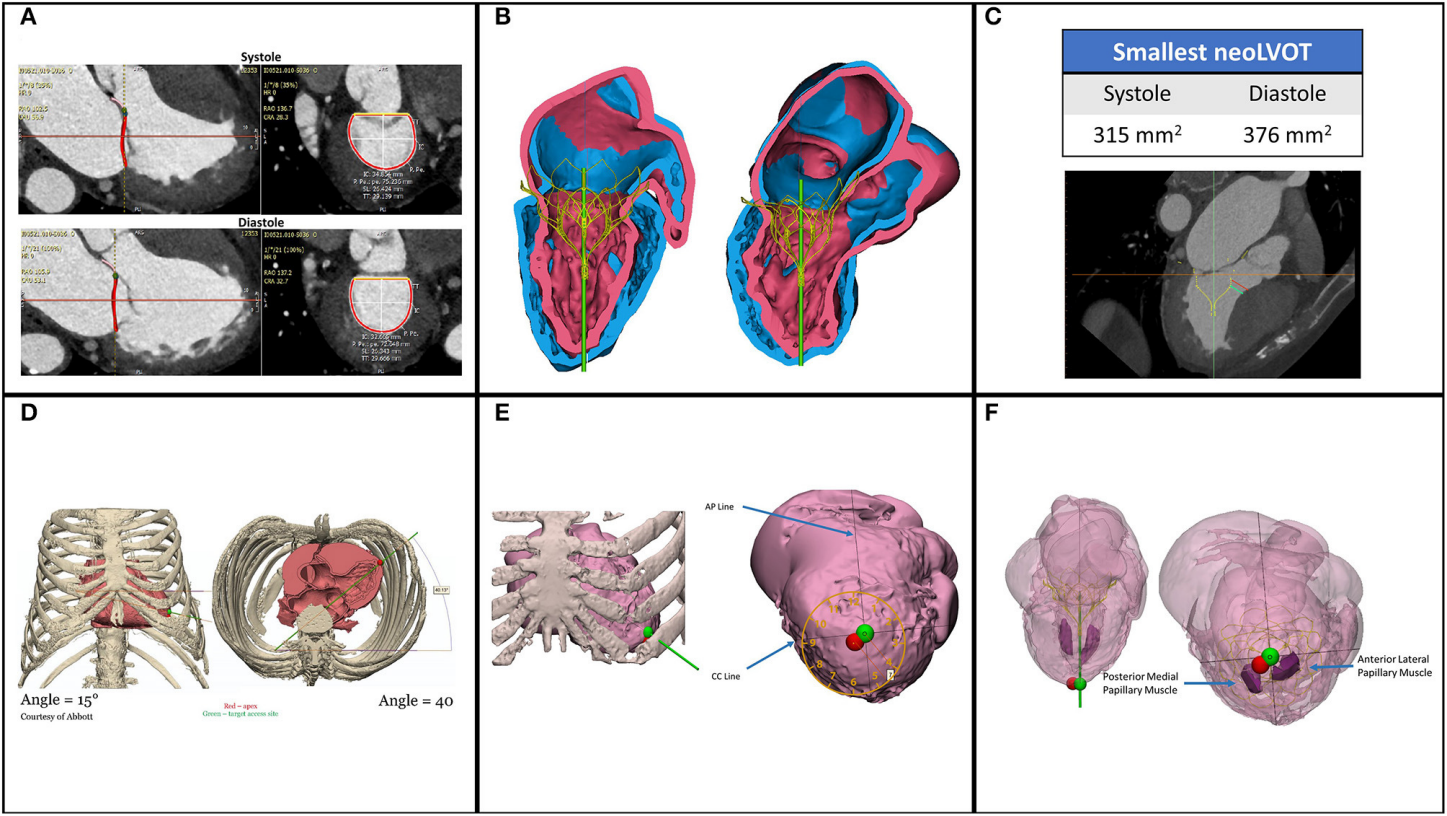
Pre-procedural planning by CT reconstructions. **(A)** Measurements for sizing are done both in systole and diastole to calculate for the best fitting. **(B)** Simulation of the selected valve to evaluate the neoLVOT and sealing of the valve **(C)**. Neo LVOT is calculated in systole and diastole. **(D)** Calculation of off table angles for delivery sheath. **(E)** The best coaxial puncture point (green) is generally slightly different from the anatomical apex **(F)**. Papillary muscles are not in the path of the delivery sheath to avoid damage. Courtesy of T Vilkama (Abbott) and P Blanke (Univ. British Columbia and St. Paul's Hospital).

Figure 4 gives a summary of pre-procedural planning.

## Sizing

**Figure 5:**
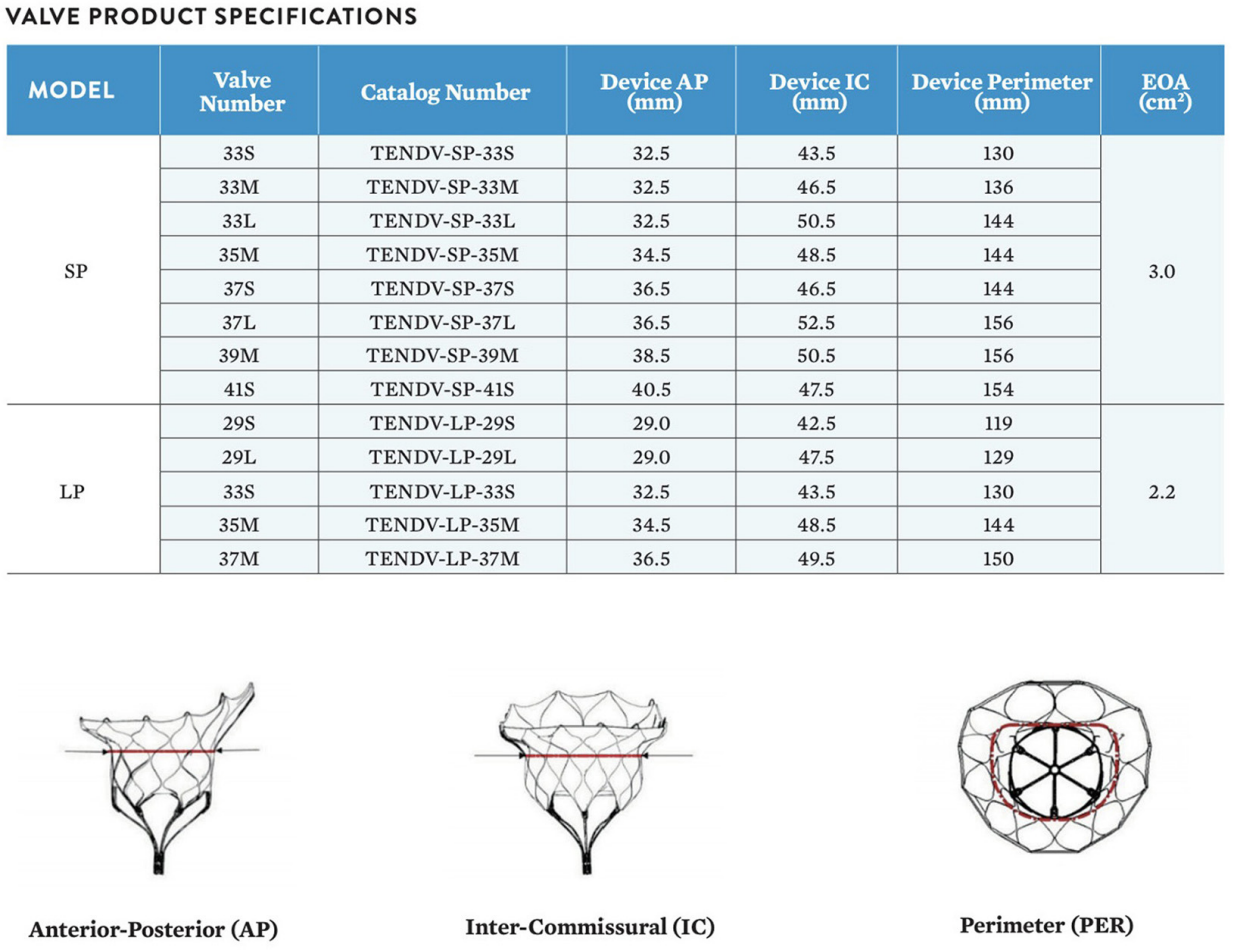
The Tendyne valve is in 13 sizes, 8 standard profile (SP) and 5 low profile (LP). The size is calculated according to the anterior-posterior (AP) diameter, the inter-commisural (IC) diameter and the perimeter (PER). Courtesy of Abbott.

The area, perimeter, septolateral, and intercommisural diameter of the mitral annulus is calculated both in diastole and systole. Sizing recommendations can be device-specific and are typically provided in the manufacturer's instructions for use. The different devices include specific sizing charts to select the right valve size according to the manufacturer's sizing calculations. For Tendyne there are currently 13 different commercially-available valve sizes, eight standard profile (SP) and five low profile (LP) sizes is according to antero-posterior diameter, inter-commissural diameter, and perimeter, Figure 5.

## The Design of Tendyne

The Tendyne mitral valve system consists of the Tendyne Mitral valve with Apical Pad and a Tendyne mitral valve delivery system. In addition, there is a retrieval system should the valve need to be retrieved during the implant procedure.

### Tendyne Mitral Valve With Apical Pad

**Figure 6:**
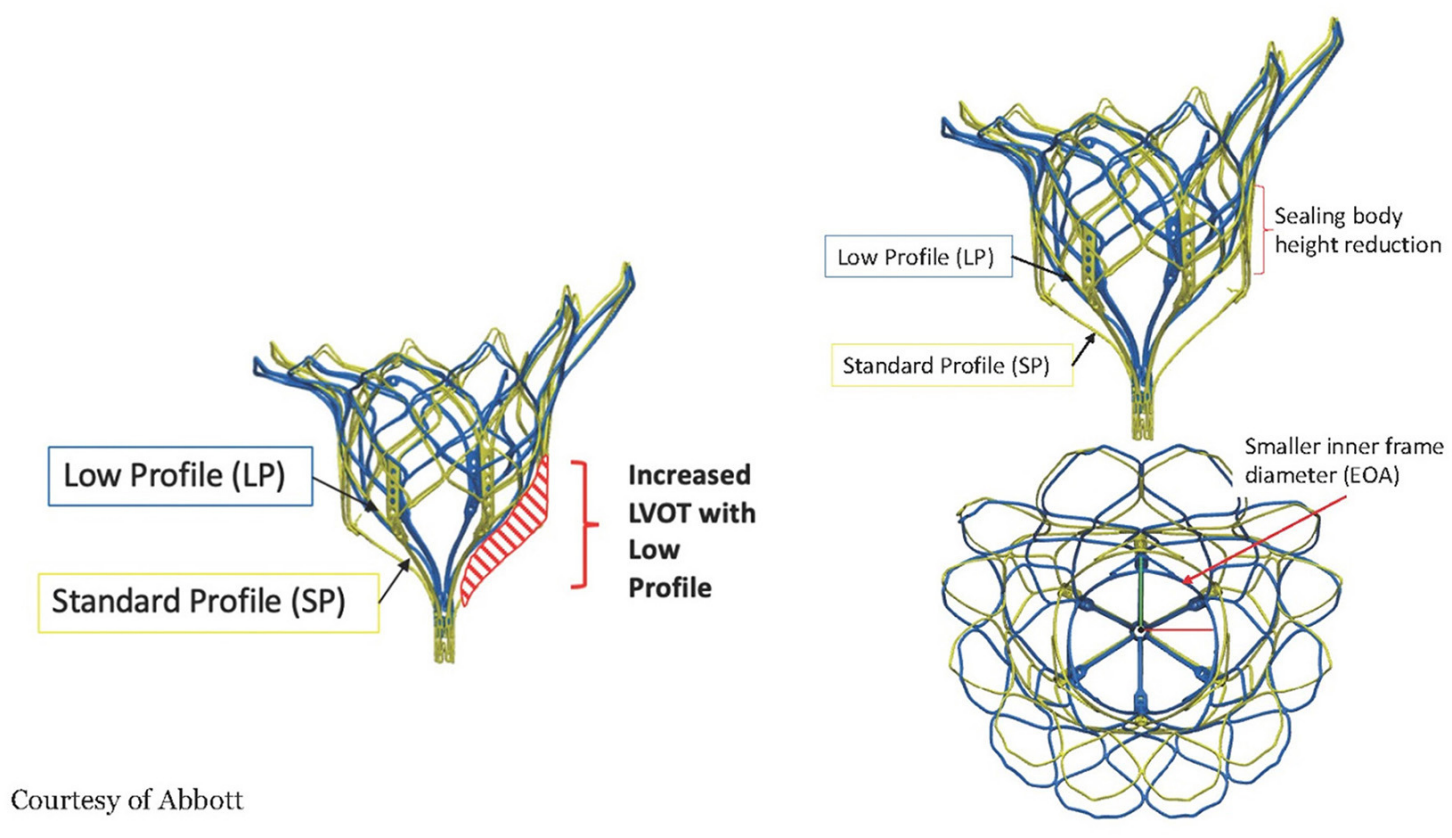
The frame of the Tendyne valve is in two designs, the Standard profile and the Low profile. The low profile enhances the neo LVOT area but also has a smaller effective orifice area and sealing body. Courtesy of Abbott.

The valve is a double frame device attached to an adjustable tether anchored to an apical pad. The inner frame supports the bioprosthetic valve and is in two sizes for either the Standard or Low Profile prosthesis families. Three porcine pericardial tissue leaflets are sewn onto a circular self-expanding nitinol frame. The inner frame is sewn inside a self-expanding nitinol outer frame covered with a polyethylene terephthalate (PET) fabric cuff, which provides the sealing surface within the native annulus. Multiple outer frame sizes are available in both a Standard Profile and a Low Profile configuration that provide different sealing height and protrusion into the LV. The Standard Profile and the Low Profile valve have an effective orifice area of ~3.0 and 2.2 cm^2^, respectively, Figure 6.

**Figure 7:**
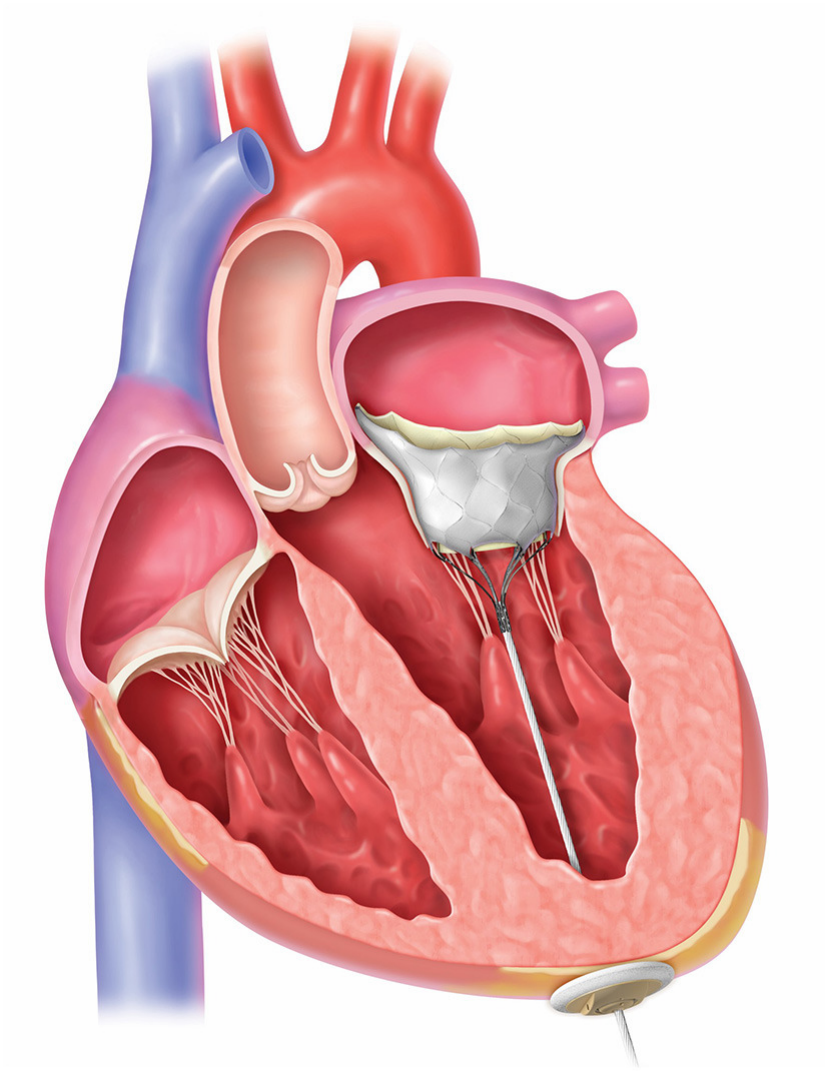
The Tendyne valve placed in the left ventricle. Courtesy of Abbott.

The outer frame is contoured to fit the native mitral anatomy and has a radiopaque marker at A1 to confirm orientation on fluoroscopy. The outer-frame cuff is raised along the A2 region and the valve is designed for radial orientation such that the anterior part of the cuff rests upon the aortomitral continuity. This orientation aligns the raised A2 region of the cuff with the anterior portion of the native mitral valve. Figure 7 shows an illustration of the Tendyne valve implanted within the mitral annulus.

**Figure 8:**
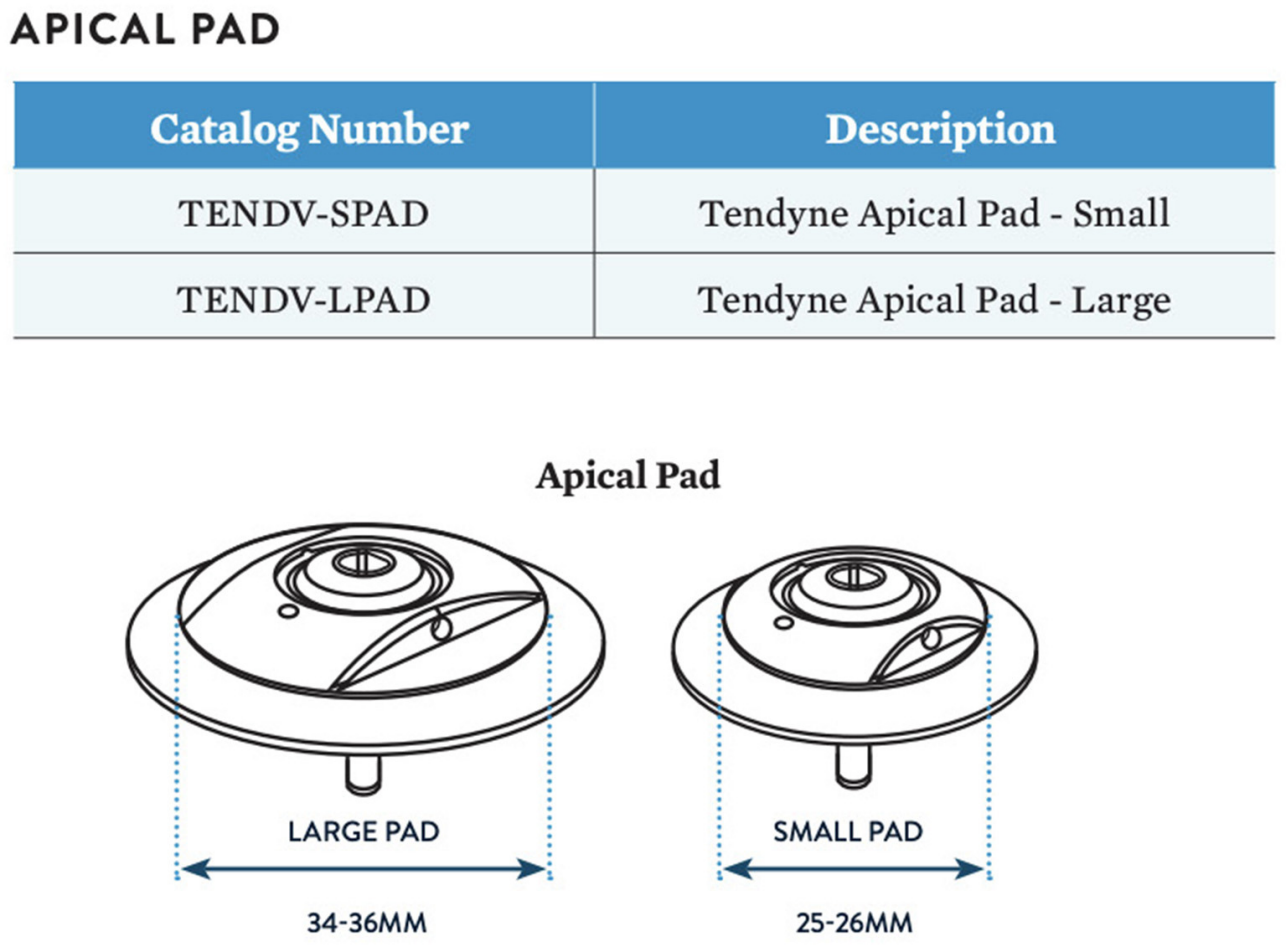
Apical pad in two sizes. Courtesy of Abbott.

The prosthesis is connected to a braided fiber tether made of high molecular weight polyethylene, which stabilizes the valve by passing through the left ventricular myocardium near the apex, where it is fastened to an Apical Pad on the epicardium. This pad is made in two sizes and is comprised of a polyether ether ketone (PEEK) button covered by a double velour polyester (PET) fabric, Figure 8.

## Tendyne Delivery System

The Tendyne system has a delivery sheath of 36 Fr for deployment of the valve. The valve is fully repositionable and retrievable intraoperatively by engaging the tether. Repositioning allows optimization of valve position following deployment. Retrieval is possible with the use of the specifically-designed retrieval system and allows use of an alternative valve size if the initial valve does not perform optimally due to selected size or position, or the neoLVOT is too small.

### Tendyne Pad Position System

The pad position system fastens the apical pad to the braided tether at the epicardial surface. It employs a Tether load system (TLS) that is used for adjusting the tensile load of the tether, thereby seating the valve within the mitral annulus. The pad is placed over the apical puncture site and may also contribute to sealing the apical access/purse strings to prevent bleeding from apex.

## Challenges To TMVI

### Challenges in Patient Selection

To date, TMVI has largely been reserved for patients who are poor candidates for surgery and where transcatheter repair is unlikely to yield durable MR reduction. Ongoing clinical trials such as SUMMIT will provide data to inform expansion of this approach into new patient populations.

The patients referred are comorbid and frail. The first clinical consideration is if the patient can tolerate a transapical intervention. Renal- and liver function are of great importance, as well as how the patient functions in daily life. Patients with heart failure (HF) should have optimal HF treatment before the procedure, including cardiac rhythm management devices such as ICD or CRT, if indicated. Other comorbidities to consider are RV dysfunction or severe tricuspid regurgitation, COPD, and ability to tolerate anticoagulation.

### Anatomical Challenges

For non-calcific MR, prosthesis sizing and annular support is crucial. The Tendyne system can be used to treat patients with annular dimensions up to 41.3 mm in the SL dimension and 143 mm in perimeter. Very small annular dimensions such as those observed in patients with severe MAC may prevent the inner frame from fully expanding and should be excluded. Conversely, patients with very large annular dimensions outside of the ranges listed by the manufacturer may be at elevated risk of PVL due to insufficient interaction between the prosthesis and the native annulus.

The shape of the Tendyne prosthesis is intended to conform to the anatomical shape of the annulus.

The goal of the implantation is to anchor the prosthesis for long-term freedom from migration, while avoiding LVOT obstruction and paravalvular leak (PVL). Proper prosthesis sizing, orientation, and tether tension work in collaboration to provide an optimal implant. In very rare instances, the Tendyne valve has been re-seated within the mitral annulus post-index procedure by adjusting the apical pad and/or tether through the original mini-thoracotomy site (12, 13).

Patients with a prior aortic valve replacement should be evaluated to ensure no interaction with TMVI prosthesis. LV chamber size must be sufficiently large for the prosthesis to allow for tether tensioning and avoid significant contact with the myocardium.

### Challenges in Sizing

The valve size is calculated using measurements taken in systole and diastole, including the perimeter, septolateral, and intra-commissural measurements. The Tendyne valve is currently available in 13 sizes with the standard profile and low profile frame, which means that that it is possible to tailor the prosthesis size specifically to many different patient anatomies. This sizing strategy is distinct from other TMVI systems that are only available in 2–4 sizes, see Table 1.

## Challenges Regarding Left Ventricular Outflow Tract (LVOT) Obstruction

LVOT is the anatomical region of left ventricle between the anterior mitral leaflet and the left ventricular septum where blood flows before reaching the aorta through the aortic valve. With the large prosthesis size of most TMVI systems, in addition to be anatomically close to the LVOT, LVOT obstruction is a large design hurdle to overcome. To avoid LVOT obstruction many factors need to be considered:

- The protrusion of the TMVI into the left ventricle
- The flaring of the prosthesis created from the anchoring method may extend to the LVOT
- The angle between the aortic and mitral planes, the aorto-mitral annular angle, will determine the protrusion of the prosthesis into the LVOT and may affect blood flow dynamics
- Septal bulging can create narrowing of the LVOT in systole
- Length of the anterior leaflet and potential for obstruction due to SAM

To evaluate these factors, CT reconstructions are done to calculate the neo-LVOT, the new LVOT area predicted after the TMVI prosthesis is implanted. An area of at least 200–250 mm^2^ is typically recommended as the cut off (14). To reduce the septal bulge it is possible to do alcohol septal ablation (15). If the anterior leaflet is too long and at prohibitive risk for SAM, the Lampoon procedure may be used to lacerate it (16) or NeoChords may be inserted to prevent anterior motion during systole.

The ability to tighten the tether under echo guidance gives the opportunity to reduce and avoid paravalvular leakage and LVOT obstruction. Intentional tether angulation may help avoid LVOT obstruction to some degree in addition to prevent migration of the Tendyne valve.

## Challenges In Delivery Method

Early TMVI designs have used the transapical approach due to the size of the valve and delivery system; however, next generation devices have had significant development efforts in employing the trans-septal approach. Still, the patients have to be in general anesthesia due to the thoracotomy for the transapical access and transesophageal guidance.

### Challenges in Re-adjustment and Retrievability

The ability to readjust and recapture the device after it has been implanted and had post-implantation performance tests conducted is valuable. In that way LVOT obstruction, unfavorable position and para valvular leak may be avoided, and better valve function obtained.

The Tendyne valve is repositionable and fully retrievable. If the valve is not seated anatomically in the annulus, the valve may be repositioned before it is fully deployed. If deployed and there is significant paravalvular leak or LVOT obstruction, the valve may be retrieved using the specially- designed retrieval system. The valve may also be retrieved if it migrates during deployment or if an alternate size is desired.

**Figure 9:**
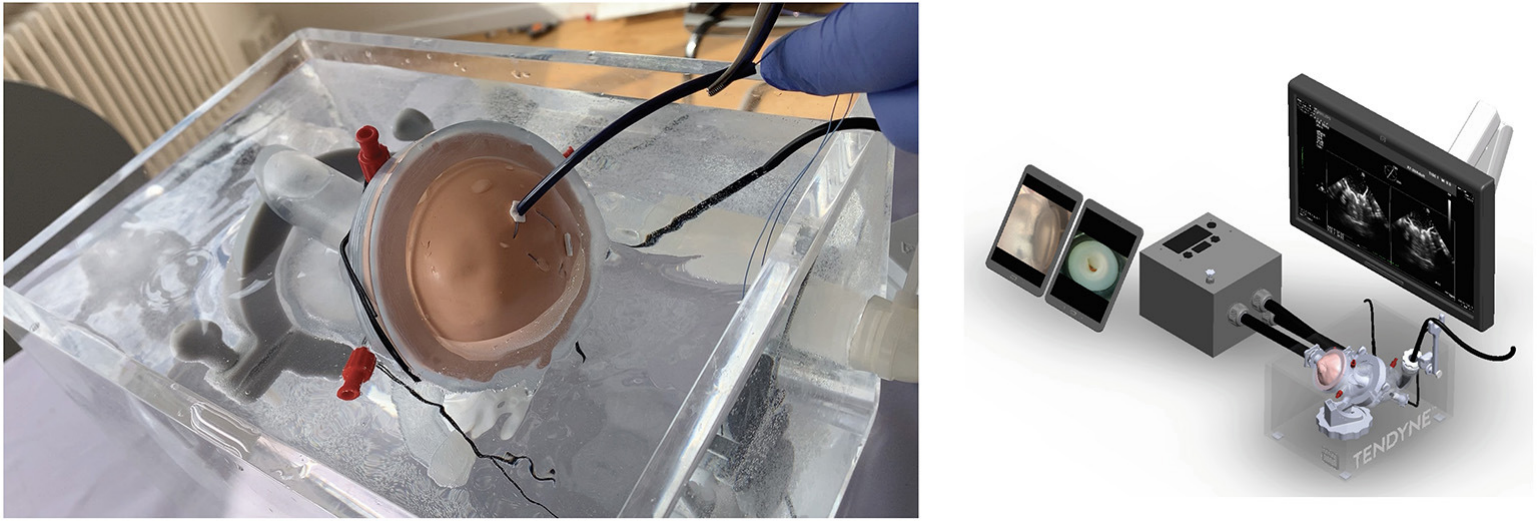
Simulator for Tendyne implant that includes a human-like 3D printed plastic mitral valve and echo imaging. Courtesy of Abbott.

A specific simulator has been designed to train for the implant. This innovation is very realistic, without use of animal models. A 3D printed mitral valve is mounted in a fluid container containing an echo probe for guidance, Figure 9. With this simulator, the operator will be able to practice the procedure and understand the technical operation of the delivery system.

### Challenges in Hemodynamics

Preoperatively it is important to optimize heart failure treatment, both for the right and left ventricle. It is also important to stabilize heart rhythm and treat AF as much as possible to limit the frequency of the episodes. If a patient has dys-synchrony, CRT (cardiac resynchronization therapy) should be evaluated. In some cases Levosimendan (Orion Pharma International, Finland) may be useful pre- and post-operatively, then often in combination with inotropy. Sometimes Milrinone (Pfizer, New York, NY, United States) may be useful preoperatively for MR patients with heart failure.

During implant the patient may become hypotensive due to the anesthesia, blood loss, LVOT obstruction, MV inflow obstruction or afterload mismatch. It is important to evaluate these options before the procedure and have adequate monitoring during implant: CVC, oxygenation, heart rate, arterial line, left ventricular catheter, Swan -Ganz catheter.

Depending on the mitral pathology, different peri-operative medical management strategies have to be chosen: for regurgitation, inotropes may be the most useful to increase heart rate and “strengthen” contractions. For mitral stenosis or a stiff ventricle, it is more important to slow heart rhythm to help ventricular filling. For all cases the avoidance of LVOT obstruction is critical. The Tendyne valve is repositionable and retrievable even after full deployment, and either action may be performed to resolve LVOT obstruction if observed.

After deployment and MR correction, the medical management strategy should be prepared to treat afterload mismatch that can occur once the low impedance regurgitant flow has been eliminated.

### Challenges Regarding Thrombosis

Compared to AVR, bioprostheses in the mitral position tend to be larger and have more synthetic material. The low flow velocities in atrial fibrillation and large atrial volumes may exacerbate stagnation of flow and the potential for thrombosis.

Bioprosthetic valve thrombosis encompasses two different entities: clinical valve thrombosis and subclinical leaflet thrombosis. Patients with clinical valve thrombosis present often with heart failure symptoms and an increase in trans- prosthetic gradients. Subclinical leaflet thrombosis is most often an incidental finding on post procedural TEE and/or CT scan (17).

Factors associated with bioprosthetic valve thrombosis include high BMI, female sex, atrial fibrillation, poor cardiac function, fluid stagnation due to big atrial volumes and calcium supplements (18).

Treatment with oral anticoagulation is recommended to prevent and to treat valve thrombosis, and NOAC (new oral anticoagulants) are not recommended or approved for TMVI systems at this time. It is still a matter of speculation if subclinical leaflet thrombosis is associated with an increased risk for thromboembolism or accelerated valve degeneration.

For the Tendyne valve, subclinical valve thrombosis and clinical thrombosis have occasionally been observed, and anticoagulation with Warfarin with INR level 2.5–3.5 is recommended at least the first 6 months. If this regimen is maintained, routine CT is not recommended for screening.

### Challenges in Durability

Several modes of bioprosthetic valve failure exist, which vary based on patient, implant position and valve characteristics. Recent EAPCI/ASC/EACTS guidelines (19) classify valve failure in four categories:

1. Structural dysfunction: calcification and leaflet tear
2. Non-structural dysfunction: pannus formation and paravalvular leak
3. Thrombus formation
4. Endocarditis

Generally, porcine valves tend to develop leaflet tear with regurgitation, whereas bovine pericardial valves are more likely to develop stenosis. The Tendyne valve is made of porcine pericardium. The early experience so far has not observed significant valve dysfunction; however, there is very limited long-term (e.g., 5-year) clinical data to draw strong conclusions.

Bioprostheses in the aortic position have better longevity compared to the mitral position. The decreased durability in the mitral position may be related to the elevated closing pressure and hence increased hemodynamic stress in the mitral position.

These challenges are to be observed as longer-term and larger patient cohorts are studied with these novel TMVI systems.

The ability to tighten the tether of the Tendyne valve to seal better may potentially help to avoid PVL. If necessary, it is possible to re-tighten the tether days to weeks after the initial implant procedure to overcome a clinically-significant PVL. Very few re-tension cases have been done to- date, but two have been reported (12, 13). It is of great importance to seal PVL to avoid heart failure, hemolysis and endocarditis.

## Tendyne In MAC

Severe mitral annular calcification (MAC) represents high operative risk, especially for atrioventricular groove rupture and severe paravalvular leak.

**Figure 10:**
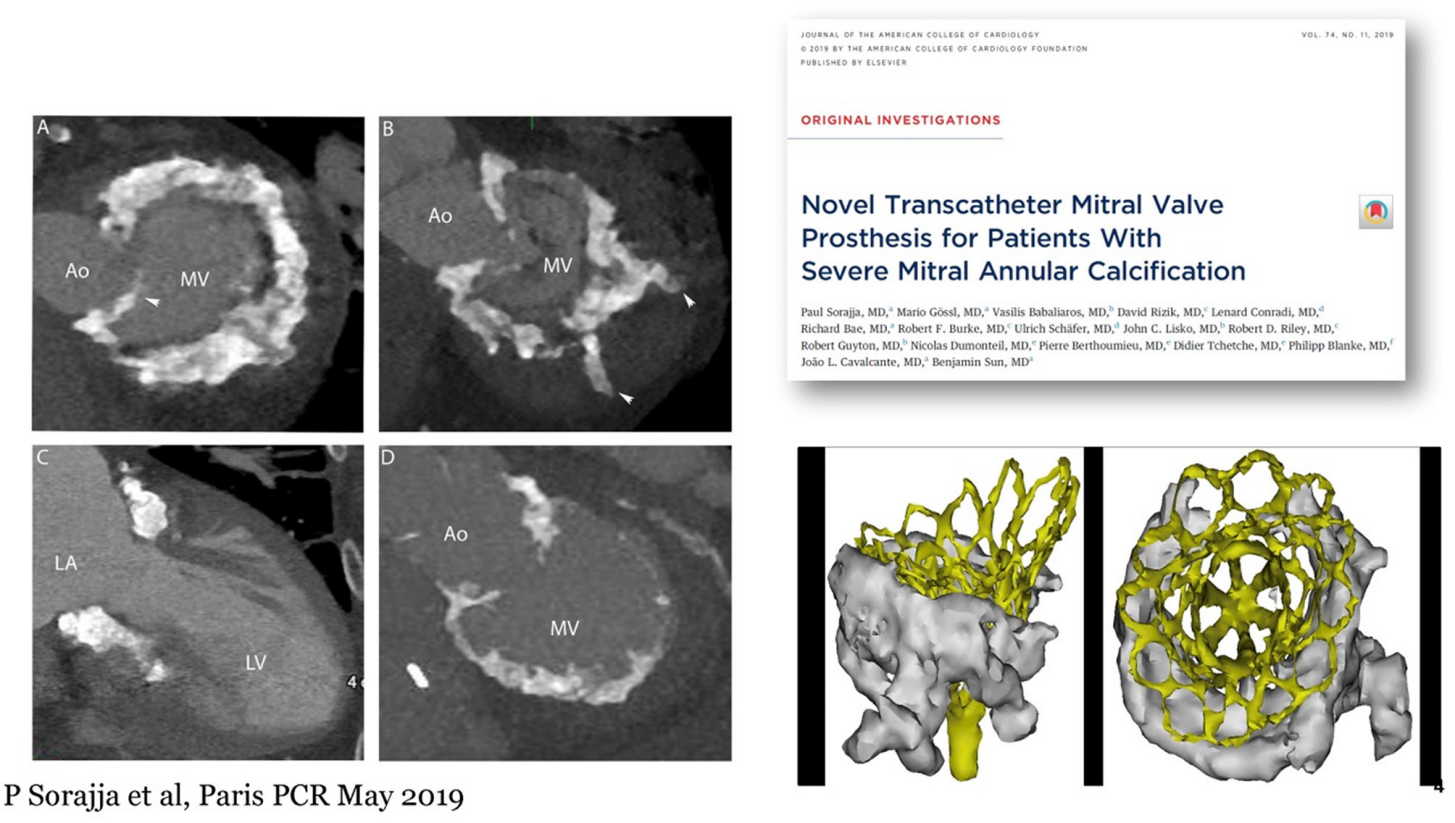
Experience of Tendyne in MAC. First Presented by Sorajja et al. (20). Left: Representative Patterns of Severe MAC in Patients With Symptomatic MR Images from gated, contrast-enhanced cardiac computed tomography are shown. **(A,B,D)** are from unique patients, whereas **(A,C)** are from the same patient. Some patients had large spicules arising from the anterior horn of the mitral annulus (**A**, arrowhead) or invading the myocardium (**B**, arrowheads). Ao, aorta; LA, left atrium; LV, left ventricle; MAC, mitral annular calcification; MR, mitral regurgitation; MV, mitral valve. Right: Tendyne implanted in MAC.

The Tendyne Mitral Valve System has successfully been implanted in nine patients with severe MAC under compassionate use (20) Figure 10. Device implantation with complete relief of MR occurred in all patients, and there were no instances of device embolization, significant mitral stenosis, or need for cardiopulmonary bypass or hemodynamic support. Technical success was achieved in eight of the nine patients. One patient without technical success had LVOT obstruction (peak gradient = 60 mmHg) due to inadvertent rotation of the prosthesis. While this patient had no residual MR, the LVOT obstruction was recognized only after surgical closure, and successful alcohol septal ablation (final peak LVOT gradient, 10 mmHg) was performed. Median follow-up for the study was 12.0 months (range, 1–28 months). One patient died in hospice on post-operative day 41. One patient, who had uncomplicated TMVI, normal prosthetic function, no recurrent MR, and no cardiac symptoms, died suddenly in an accident unrelated to the prosthesis 8 months after the procedure. All other patients survived to the end of follow-up. MR remained absent in all treated patients. There was no evidence of prosthesis dysfunction, including no significant mitral stenosis (mean gradient, 3.4 ±1.8 mmHg), hemolysis, thrombosis, as well as no major adverse clinical events in follow-up.

**Figure 11:**
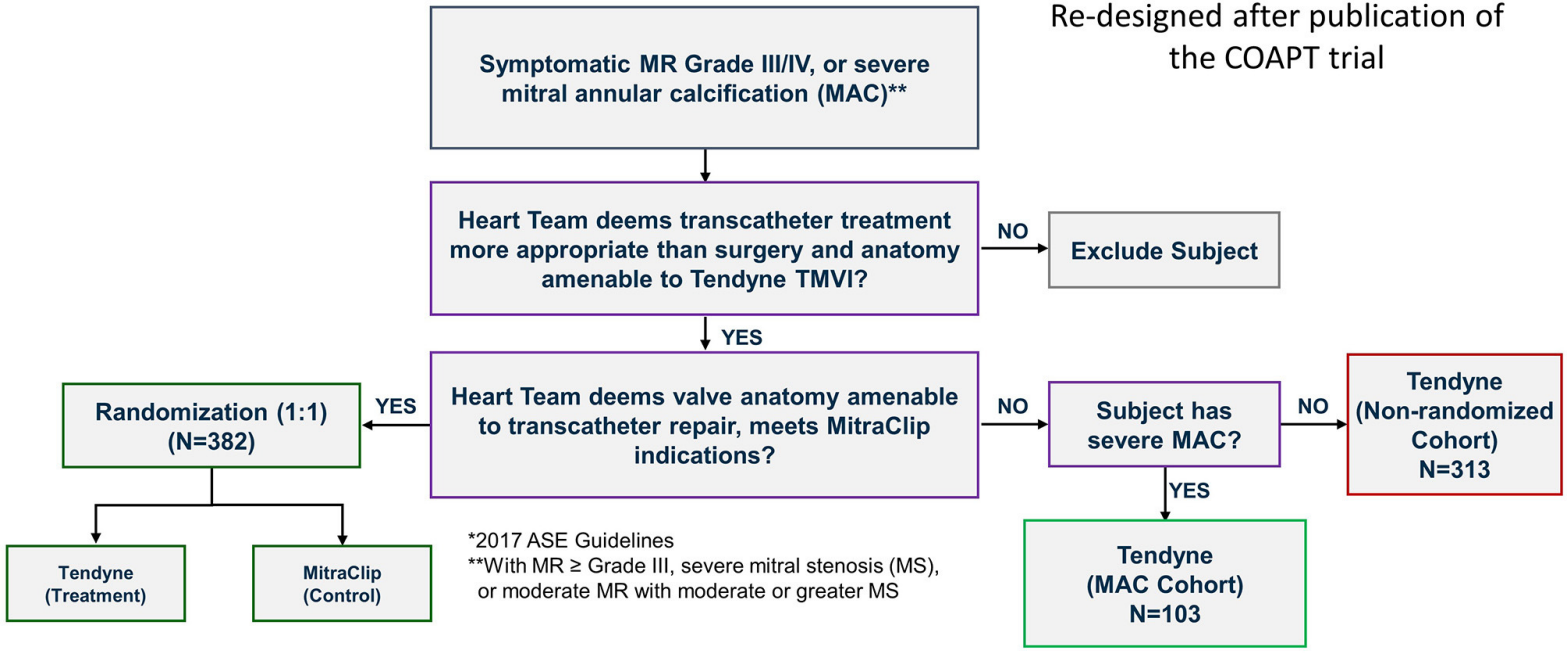
The SUMMIT trial design that includes randomization between Tendyne and MitraClip. Separate cohorts enroll patients with severe MAC or are otherwise not suitable for MitraClip and therefore are in the single-arm non-randomized cohort where all subjects are treated with Tendyne. Courtesy of Abbott.

The positive experience in these patients motivated Abbott to develop a formal Feasibility Study of up to 30 patients that is ongoing in the US. In the SUMMIT trial there is a special arm investigating this challenging patient population, Figure 11.

Eleven patients were treated in the Feasibility Study, which is now closed for enrollment due to MAC patients being included in the larger SUMMIT trial. To date, there has been one death within 30 days of the procedure in the combined compassionate use and MAC feasibility study experience (1/20, 5%). Though the current experience in patients with severe MAC is limited, these early outcomes compare extremely favorably to those reported with off-label use of balloon-expandable aortic prostheses in MAC, where 30-day mortality was 25% (21).

Recently, Guerrero et al. published an article on CT evaluation and classification of MAC to predict embolization of balloon-expandable valves when implanted in MAC (22).

## Tendyne With Previous AVR/TAVI

**Figure 12:**
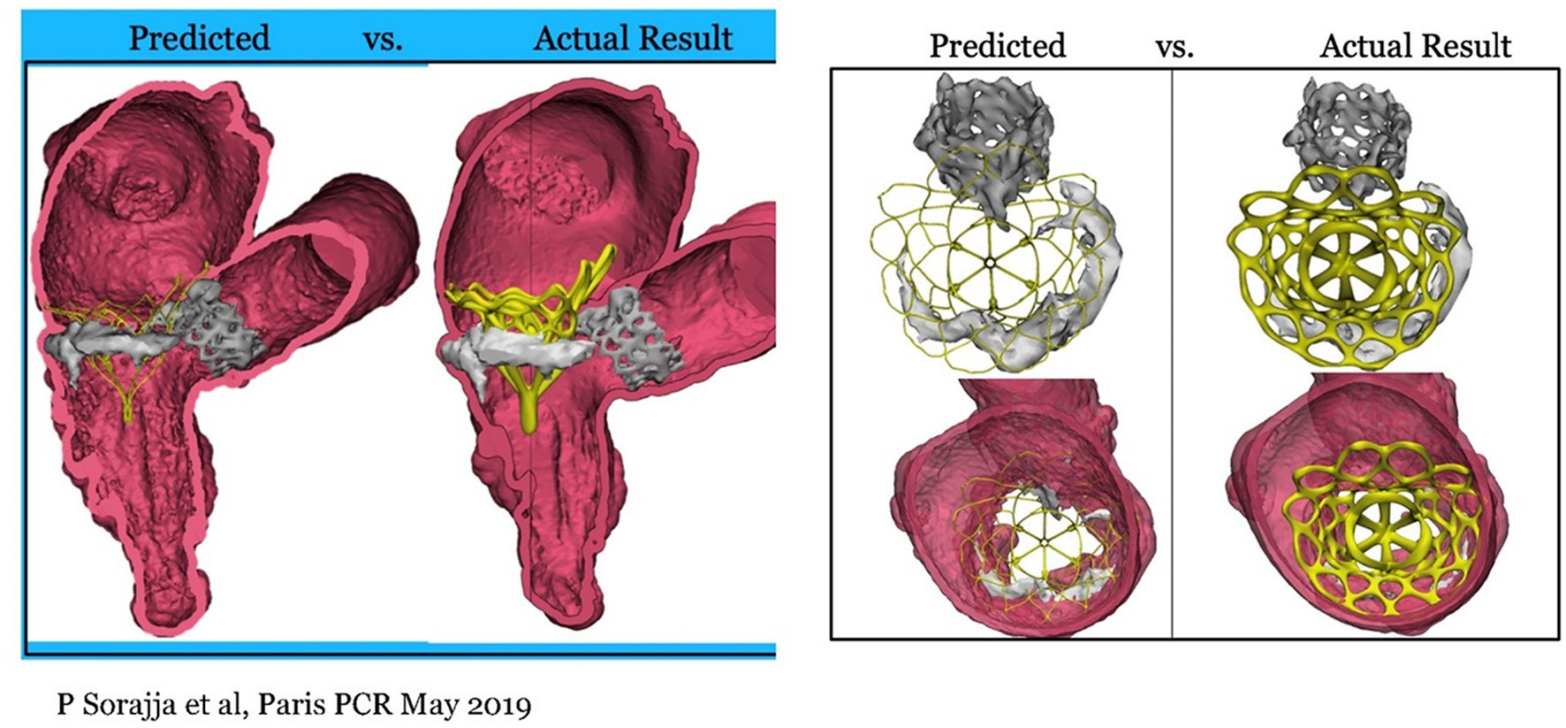
Tendyne concomitant to AVR and TAVI. First experience presented at Paris PCR 2019 (23).

In surgical mitral valve replacement, a preexisting aortic valve implant (AVR/TAVI) may cause difficulties in replacing the mitral valve, and often the aortic prosthesis has to be removed and re- implanted after the mitral valve replacement. It has been feasible to implant a Tendyne with anaortic prosthesis in place, either surgical or transcatheter, without altering the function of the prosthesis (23), Figure 12.

## Summary

The Tendyne valve is the only CE approved transcatheter mitral valve implant. The prosthesis is repositionable and retrievable and this feature may help to avoid LVOT obstruction and also PVL, two of the main obstacles for TMVI. Tendyne is available in a large number of sizes that can cover a large range of annular dimensions. Coumadin postoperatively may help avoid valve thrombosis and long- term degeneration, which will continue to be evaluated with longer-term data in larger patient cohorts.

## Data Availability Statement

Publicly available datasets were analyzed in this study. This data can be found at the following places: https://doi.org/10.1016/j.jacc.2016.10.068, https://doi.org/10.1016/j.jacc.2018.12.066, https://doi.org/10.1016/j.jcin.2020.05.010, https://doi.org/10.1016/j.jacc.2019.07.069, https://pubmed.ncbi.nlm.nih.gov/32718908.

## Ethics Statement

The studies involving human participants were reviewed and approved by multiple committees. The patients/participants provided their written informed consent to participate in this study.

## Author Contributions

The author confirms being the sole contributor of this work and has approved it for publication.

## Conflict of Interest

The author declares that the research was conducted in the absence of any commercial or financial relationships that could be construed as a potential conflict of interest.
